# Draft Genome Assemblies of Clinical Isolates of Klebsiella pneumoniae V9011662 and Enterobacter hormaechei Entb306

**DOI:** 10.1128/MRA.00091-19

**Published:** 2019-04-11

**Authors:** Lucas B. Harrison, Anna Selmecki, Nancy D. Hanson

**Affiliations:** a Department of Medical Microbiology and Immunology, Creighton University, Omaha, Nebraska, USA; University of Maryland School of Medicine

## Abstract

Enterobacter hormaechei and Klebsiella pneumoniae are pathogenic Enterobacteriaceae that have been associated with the spread of antibiotic resistance. Here, we report draft genome assemblies of an Enterobacter hormaechei clinical isolate and a multidrug-resistant clinical isolate of Klebsiella pneumoniae.

## ANNOUNCEMENT

Klebsiella pneumoniae and Enterobacter hormaechei strains harboring antibiotic resistance genes pose a threat to human health (1, 2). Urinary tract infections caused by these organisms run the risk of progressing to urosepsis, and antibiotic resistance genes can increase the risk of clinical complications (2–4). This genome announcement presents draft genome sequences of pathogenic clinical human isolates of K. pneumoniae strain V9011662, obtained from a diagnostic repository, and E. hormaechei strain Entb306, collected from a male patient in Kentucky.

Overnight cultures were grown at 37°C under agitation in Mueller-Hinton broth. DNA was harvested using the Qiagen DNeasy blood and tissue DNA extraction kit and purified using an Amicon Ultra centrifugal filtration column. Illumina Nextera XT DNA sequencing libraries were prepared with version 3 technology and optimized for 300-bp paired-end reads (5). Low-quality reads were filtered from the sequencing data using Trimmomatic version 0.33 and screening for TruSeq3 adapter sequences. In Klebsiella pneumoniae, the filtered sequence library consisted of 3,799,690 read pairs and 1,100,940 singlets. The Enterobacter hormaechei filtered sequence library consisted of 4,657,517 read pairs and 723,499 singlets.

The genomes were assembled as previously described (6). Contig libraries generated from the SPAdes version 3.10 and SGA version 0.10.15 assemblers were combined to create an initial *de novo* assembly (7, 8). SPAdes was run using the parameters “-k 21,33,55,77,99,127,” “-careful,” and “–cov-cutoff 5.0.” SGA was run using default parameters, ensuring that the “–pe-mode” flag was set to 1. Contigs were mapped to closely related genomes using Ragout version 1.2, with default settings (GenBank accession numbers NC_009648, NZ_CP009775, and CP011989 for K. pneumoniae and CP010384, CP008823, and FP929040 for E. hormaechei) (9). AlginGraph was used for gap closure, with the “distanceLow” and “distanceHigh” parameters set to 50 and 1,602, respectively. The assembly sequences were improved by iCORN2 using default parameters (10, 11). This assembly was then used as the reference genome in generating final *de novo* assemblies with SPAdes.

The K. pneumoniae assembly for V9011662 was 5,708,850 bp long, containing genomic and plasmid DNA in 63 contigs, with a mean read coverage of 91.52×. The assembly had a GC content of 57.60%, with *N*_50_ and *N*_75_ values of 223,468 and 100,593, respectively, and *L*_50_ and *L*_75_ values of 7 and 16, respectively. This assembly contained 5,891 genes encoding 5,632 protein-coding regions, 80 tRNAs, 11 noncoding RNAs (ncRNAs), and 147 pseudogenes, as determined by the NCBI Prokaryotic Genome Annotation Pipeline. The plasmid replicon sequence database from PlasmidFinder was downloaded, and the assembled sequence was queried against the database using BLASTn. PlasmidFinder screening for replicon sequences detected IncX3, IncFII, IncFIB, and ColRNAI plasmid replicons, while ResFinder detected the resistance genes *aac(6′)-Ib*, *aadA17*, *aph(3′)-Ia*, *bla*_KPC-2_, *bla*_TEM-1A_, *bla*_OXA-9_, *bla*_SHV-182_, *aac(6′)-Ib-cr*, *oqxA*, *oqxB*, *fosA*, *mph*(A), *catA1*, *sul1,* and *dfrA12* (12).

The E. hormaechei assembly for Entb306 was 4,861,244 bp long, containing genomic DNA in 7 contigs, with a mean read coverage of 79.3×. This assembly had a GC content of 55.5%, with *N*_50_ and *N*_75_ values of 2,519,539 and 691,652, respectively, and *L*_50_ and *L*_75_ values of 1 and 3, respectively. This assembly contained 4,711 genes encoding 4,590 protein-coding regions, 93 tRNAs, 6 ncRNAs, and 72 pseudogenes, as determined by the NCBI Prokaryotic Genome Annotation Pipeline. ResFinder detected two resistance genes, *bla*_ACT-16_ and *fosA*.

E. hormaechei and K. pneumoniae are organisms of concern for their ability to transmit antibiotic resistance genes. Here, we provide two draft genome sequences of E. hormaechei and K. pneumoniae clinical isolates. One, that of Entb306, contains only two antibiotic resistance genes, making it useful for reference assemblies. The other, V9011662, contains multiple plasmids, which results in a library of resistance genes. Understanding genomic mechanisms of antibiotic resistance is key to developing new strategies to combat this global threat, and the addition of clinical isolate draft genome sequences will further this aim.

### Data availability.

These whole-genome shotgun projects have been deposited at DDBJ/ENA/GenBank under the accession numbers RXFU00000000 and RXFV00000000 for E. hormaechei Entb306 and K. pneumoniae V9011662, respectively. The versions described in this paper are RXFU01000000 and RXFV01000000, respectively, with SRA accession number PRJNA508973.
